# Impaired white matter integrity between premotor cortex and basal ganglia in writer’s cramp

**DOI:** 10.1002/brb3.1111

**Published:** 2018-09-21

**Authors:** Maria Berndt, Yong Li, Gina Gora‐Stahlberg, Angela Jochim, Bernhard Haslinger

**Affiliations:** ^1^ Department of Neurology, Klinikum rechts der Isar Technische Universität Muenchen Muenchen Germany; ^2^ Department of Neuroradiology, Klinikum rechts der Isar Technische Universität Muenchen Muenchen Germany

**Keywords:** diffusion tensor imaging, dystonia, fiber tracking, Writer’s cramp

## Abstract

**Introduction:**

Writer’s cramp (WC) as a focal hand dystonia is characterized by abnormal postures of the hand during writing. Impaired inhibition and maladaptive plasticity in circuits linking the basal ganglia and sensorimotor cortices have been described. In particular, a dysfunction of lateral premotor cortices has been associated with impaired motor control in WC. We applied diffusion tensor imaging to identify changes in white matter connectivity between premotor regions and important cortical and subcortical structures.

**Methods:**

Whole brain white matter tracts were reconstructed in 18 right‐handed WC patients and 18 matched controls, using probabilistic fiber tracking. We restricted our analyses to left‐hemispheric fibers between the middle frontal gyrus (MFG) and basal ganglia, thalamus, primary motor, and sensory cortex. Diffusion parameters (fractional anisotropy and linear anisotropy) were compared between both groups.

**Results:**

A significant reduction in fractional anisotropy values was shown for patients (mean ± *SD*: 0.37 ± 0.02) vs. controls (0.39 ± 0.03) regarding fibers between the left‐sided MFG and the putamen (*p* < 0.05). The same applied for linear anisotropy values in this connection (*p* < 0.05).

**Conclusions:**

Our results suggest an impaired structural connectivity between the left‐hemispheric MFG and putamen with a loss of equally aligned fibers in WC patients. This could reflect a structural basis for functional findings interpreted as altered inhibition and plasticity, both within the premotor cortex and the basal ganglia, that at last lead to the clinical symptoms of WC.

## 1 INTRODUCTION

Writer's cramp (WC) is an isolated idiopathic task‐specific focal hand dystonia (Albanese et al., 2013; Epidemiological Study of Dystonia in Europe Collaborative, 2000), leading to involuntary hyperactive contractions and dystonic postures of hand and arm muscles during writing only (simple WC) or also during other hand motor tasks (dystonic WC; Schenk, Bauer, Steidle, & Marquardt, 2004).

The pathophysiology of idiopathic dystonia is not entirely understood yet (Breakefield et al., 2008). Several imaging approaches have been applied to explore intracerebral changes in dystonia, the majority in WC. Mainly using positron emission tomography (PET) and functional magnetic resonance imaging (fMRI), most WC studies showed dysfunctional activation patterns in cortical regions, especially in somatosensory, primary motor, and premotor cortices, as well as in the basal ganglia and the thalamus (Castrop, Dresel, Hennenlotter, Zimmer, & Haslinger, 2012; Ceballos‐Baumann, Sheean, Passingham, Marsden, & Brooks, 1997; Ibanez, Sadato, Karp, Deiber, & Hallett, 1999; Oga et al., 2002). The functional connectivity between these areas was shown to be altered in a resting‐state fMRI analysis (Dresel et al., 2014).

By contrast, imaging‐based analyses of structural changes in WC are much less numerous so far. Morphometric studies described changes in gray matter density especially in the basal ganglia, thalamus, as well as prefrontal and sensorimotor cortex (Delmaire et al., 2007; Egger et al., 2007; Garraux et al., 2004). White matter tracts can be reconstructed by diffusion tensor imaging (DTI) MR techniques. DTI studies in generalized dystonia have contributed substantially to the present concept of a network disorder including the sensorimotor cortex, thalamus, basal ganglia, brainstem, and cerebellum (Argyelan et al., 2009; Neychev, Gross, Lehericy, Hess, & Jinnah, 2011; Niethammer, Carbon, Argyelan, & Eidelberg, 2011). The majority of DTI studies in focal dystonia have been performed using voxelwise measurements of DTI scalars such as fractional anisotropy (FA) or mean diffusivity (MD; Bonilha et al., 2007; Colosimo et al., 2005; Fabbrini et al., 2008; Prell et al., 2013). In contrast, tractography directly allows analyzing fiber tract characteristics linking functional or anatomic regions of interest. Deterministic tractography showed diffusion abnormalities of fiber tracts connecting the primary sensorimotor cortex with the brainstem in WC patients (Delmaire et al., 2009). The advanced technique of probabilistic tractography offers the advantage to estimate the distribution of possible pathways between complex regions of high uncertainty such as crossing fibers. However, this has rarely been applied in dystonia, not yet in patients with WC, so far (Argyelan et al., 2009; Blood et al., 2012; Bonilha et al., 2009; Rozanski et al., 2014).

To be able to better correlate the abovementioned functional deficits to changes in structural network integrity, we aimed at exploring the integrity of fiber tracts between regions thought to be centrally involved in the pathophysiology of WC. In particular, the dorsal premotor cortex (PMC) and its connections to primary sensorimotor cortices and basal ganglia have been described to be involved in a functional lack of cortical inhibition (Hallett, 2011; Koch et al., 2008; Murase et al., 2000) and a relative overactivity of the direct pathway in WC (Hallett, 2006, 2011 ). We therefore performed probabilistic fiber tracking between the middle frontal gyrus (MFG; harboring the PMC) and important cortical and subcortical regions including the primary sensorimotor cortex, the basal ganglia, and the thalamus. We focused on left‐hemispheric connections in right‐handed subjects.

## 2 MATERIALS AND METHODS

### 2.1 Subjects

Eighteen patients with right‐sided WC (eight females, mean age 49.2 ± *SD* 14.5 years) and eighteen controls (eight females, mean age 49.9 ± *SD* 15.1 years), all of them at a maximum age of 70 years and matched for age and gender, were included in the study. All subjects were right‐handed, verified by the Edinburgh Handedness Inventory (Oldfield, 1971), and had no family history of dystonia as well as no other neurological diseases or previous neuroleptic medication. The patients were recruited from our outpatient clinic for movement disorders. Mean symptom duration was 15.2 years (*SD* ± 11.1 years), and nine patients suffered from simple or dystonic WC, respectively. Six patients were botulinum toxin (BTX)‐naive, 12 had received BTX over a period of 42.9 ± 62.1 months, and all of them at least 3 months (39.7 ± 41.1 months) prior to imaging.

All participants gave written informed consent. The study was approved by the local ethics committee at the Klinikum rechts der Isar of the Technical University of Munich, Germany, in accordance with the ethical standards of the 1964 Declaration of Helsinki and its later amendments.

### 2.2 Data acquisition

Diffusion tensor images were acquired with an eight‐channel head coil on a 3 T Achieva MRI scanner (Philips Medical System, the Netherlands) using a single‐shot spin‐echo EPI sequence with the following parameters: TE = 92 ms; TR = 11–22 beats (cardiac gated, optimal TR according to subject’s heart rate); number of diffusion directions = 64; *b*‐value = 1,400 s/mm2; flip angle = 90°; FoV (mm): 232 × 232 × 132; voxel size (mm): 1.81 × 1.81 × 2; axial slices per volume = 66. The 64‐gradient direction diffusion‐weighted (DW) images were recorded for each subject. To minimize the probability of head motion artefacts due to relatively long MRI scan duration, image acquisition was divided into two separate runs, each resulting in 32 DW images and one non‐diffusion‐weighted image (*b*‐value = 0 s/mm^2^, the average of six single b0 images).

Additionally, for anatomical reference a 3D gradient echo T1‐weighted image was acquired for all subjects using the following parameters: TR/TE = 9 ms/4 ms; flip angle 8°; matrix: 240 × 240 × 170; FoV (mm): 240 × 240 × 170; voxel size (mm): 1 × 1 × 1; slice thickness: 1 mm without a gap.

### 2.3 DTI preprocessing

Diffusion‐weighted data were processed using ExploreDTI (v4.8.3; Leemans, Jeurissen, Sijbers, & Jones, 2009) applying the following three steps: (a) correction for subject motion and eddy current‐induced geometrical distortions (Leemans & Jones, 2009), and EPI/susceptibility distortions correction (Irfanoglu, Walker, Sarlls, Marenco, & Pierpaoli, 2012); (b) calculation of the diffusion tensors using a nonlinear regression procedure (Basser & Pierpaoli, 1996); and (c) all DW data were visually screened to check the quality of tensor estimation and registration.

### 2.4 White matter tractography

For each individual dataset, white matter tractography was applied in ExploreDTI (v4.8.3; Leemans, et al., 2009) using a probabilistic tracking approach. The fibers were reconstructed applying a wild bootstrap streamline tracking algorithm that combines a bootstrap method estimating the probability density function (PDF; Pajevic & Basser, 2003) with tractography, more precisely described in Ref. (Jones, 2008). To avoid incomplete fiber reconstructions, a whole brain tractography was performed using a brute‐force approach (Huang, Zhang, Zijl, & Mori, 2004). Before region of interest (ROI)‐based fiber tracking analyses, all ROIs were aligned to the native space of all individuals using affine and elastic registration based on “elastix” (Klein, Staring, Murphy, Viergever, & Pluim, 2010) by employing the automated atlas‐based analysis function of ExploreDTI (Leemans, et al., 2009). ROIs were derived from the Automated Anatomical Labeling atlas (AAL; Tzourio‐Mazoyer et al., 2002) of the WFU PickAtlas (https://www.nitrc.org/projects/wfu_pickatlas).

### 2.5 Structural connectivity analyses

For each subject, reconstructed fibers between pairs of ROIs that were applied to extract all fiber tracts between these two brain regions, respectively, were characterized by diffusion parameters like fractional anisotropy (FA) and linear anisotropy (CL) values, averaged over all voxels passed by the fibers. These structural connectivity analyses were performed using ExploreDTI (v4.8.3; Leemans, et al., 2009). As above mentioned, left‐sided MFG was set as main seed ROI, and we analyzed its connections to ipsilateral cortical and subcortical located ROIs including the primary motor and somatosensory cortex, the basal ganglia (caudate nucleus, putamen, and pallidum), as well as the thalamus. In a second step, mean value comparisons of FA and CL values between patients and controls were made by the means of a two‐sample *t* test for independent samples using IBM SPSS Statistics version 21.0 (SPSS Inc., IBM Corp., Armonk, NY). The *p*‐values were corrected for multiple comparisons using the conservative Bonferroni correction (* = significant after correction; significance level of 0.05/6 = 0.0083) within FA and CL analysis, respectively. Because dependency exists between the scalar parameters FA and CL, CL analysis was made in addition to FA analysis to confirm the results in a descriptive way without need to correct for testing of two different variables.

As a post hoc analysis, we checked the anatomical connections, which showed differences between patients and controls within the left hemisphere, also for group differences in the right hemisphere, that is contralateral to the nondominant and clinically not affected hand (see Supporting Information Appendix S1).

## 3 RESULTS

### 3.1 Patients

Immediately before the scan, all patients were videotaped and the severity of dystonia was rated using the Arm Dystonia Disability Scale (ADDS; Fahn, 1989; mean 56.1% ± 16.5%) and Writer's Cramp Rating Scale (WCRS; Wissel et al., 1996; writing movement score [mean 13.5 ± 5.9] and writing speed [mean 1.3 ± 0.6]).

### 3.2 FA testing

**Figure 1:**
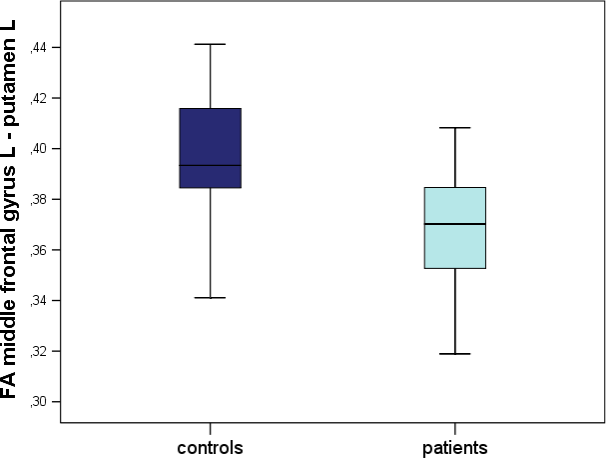
Boxplots of fractional anisotropy (FA) values of the fibers between left‐sided middle frontal gyrus and putamen (controls on the left, patients on the right)

**Figure 2:**
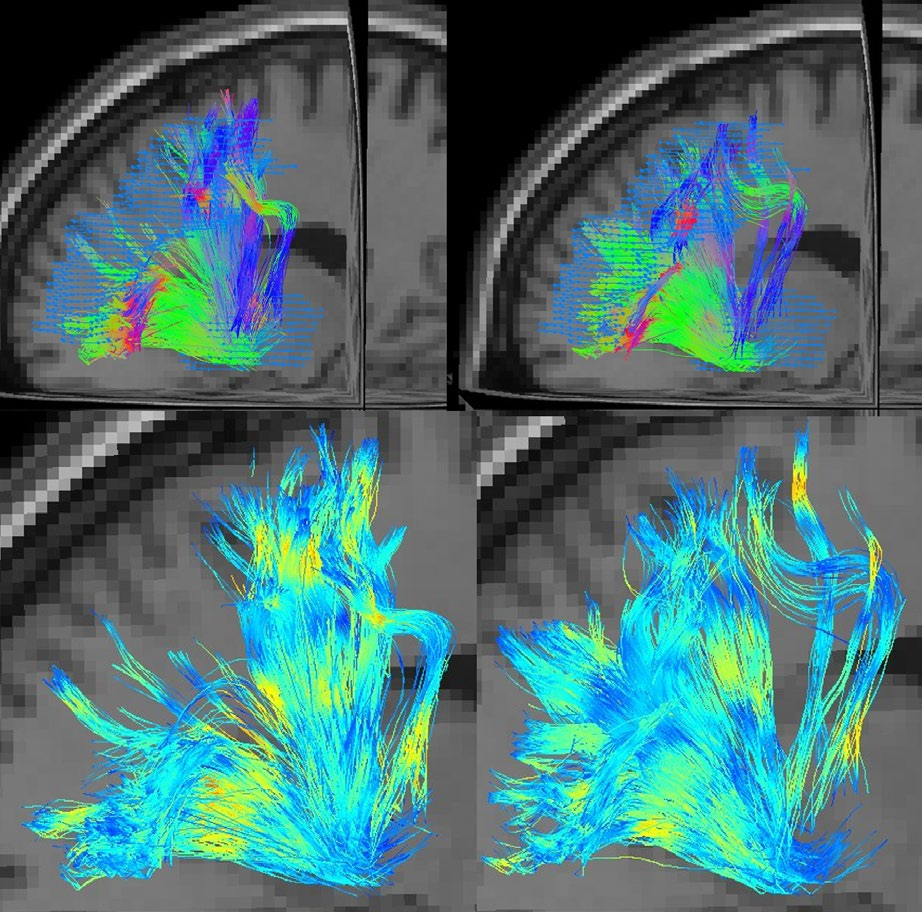
All fiber tracts between left‐sided middle frontal gyrus and putamen for controls (left) and patients (right) using DEC color coding for fibers (upper row) and voxelwise FA value coding (lower row, color‐coded from blue [FA = 0] to red [FA = 1])

When compared to controls, patients showed reduced FA values for the left‐hemispheric fibers between the MFG and the putamen (*p = 0.008), as shown in Figure 1. Figure 2 visualizes fibers of this connection for all patients and controls, respectively, in directionally encoded color (DEC) maps (Pajevic & Pierpaoli, 1999) as well as coded with voxelwise FA values. Visually, patients present more reconstructed fibers in connection with the more rostral parts of the MFG than controls, whereas less fibers are found between more caudal parts of the MFG, harboring the PMC, and putamen. It is also noticeable that all reconstructed fibers connect the anterior parts of the putamen with the MFG, whereas no fibers could be reconstructed with the posterior part of the putamen. Based on the color scale (see Figure 2), patients’ fibers exhibit lower FA values along their course, in accordance with the statistical analysis. It seems that patients’ fibers miss the FA value peak in the white matter bordering the premotor cortical structures that can be seen for controls.

All further tested fiber connections showed no statistically significant differences in the FA values between patients and controls after correction for multiple comparisons (see Table 1).

**Table 1:** Statistical parameters of the FA analysis

Connection	FA controls		FA patients		*p*‐Value (* = significant after correction)
MFG L	Mean	*SD*	Mean	*SD*	
Putamen L	0.393	0.028	0.369	0.023	0.008*
Pallidum L	0.398	0.025	0.376	0.022	0.012
Caudate L	0.360	0.054	0.351	0.035	0.677
Thalamus L	0.408	0.031	0.409	0.028	0.921
Precentral L	0.426	0.023	0.412	0.022	0.062
Postcentral L	0.424	0.024	0.411	0.032	0.172

Note

Mean and standard deviation (*SD*) of the fractional anisotropy (FA) for the tested connections with the middle frontal gyrus (MFG) and *p*‐values of the mean value comparison

### 3.3 CL testing

**Figure 3:**
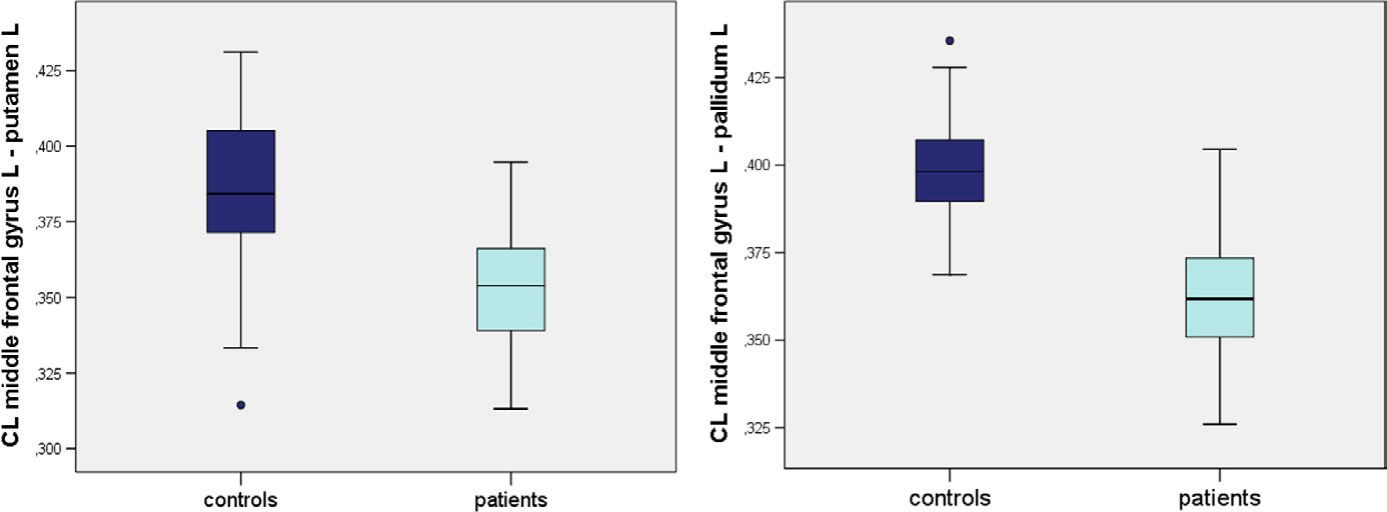
Boxplots of linear anisotropy (CL) values of the fibers between left‐sided middle frontal gyrus and putamen/pallidum (controls on the left, patients on the right)

When compared to controls, patients showed reduced CL values for the left‐hemispheric fibers between the MFG and the putamen (*p = 0.003) as well as between the MFG and the pallidum (*p < 0.001; Figure 3).

All further tested fiber connections showed no statistically significant differences in the CL values between patients and controls after correction for multiple comparisons (see Supporting Information Table S1).

### 3.4 Post hoc analysis

Based on the abovementioned results for the left hemisphere, testing of the fiber connections between the MFG and the putamen as well as the pallidum in the right hemisphere showed no significant group differences (see Supporting Information Appendix S1).

## 4 DISCUSSION

Patients with WC showed an alteration of diffusivity within the fibers connecting left‐hemispheric cortical structures harboring the PMC to the basal ganglia. FA values were reduced for the fiber tracts between MFG and putamen, suggesting an impaired structural connectivity of these regions. This result was confirmed by the CL analysis showing differences in the same connection and additionally for fibers between MFG and pallidum.

### 4.1 Interpretation of the diffusion abnormalities

In our study, we characterized reconstructed fiber tracts by diffusion parameters such as FA measuring tissue anisotropy or CL describing the linear extension of the diffusion tensor. Both parameters represent the integrity of white matter with higher values for directed diffusion in case of equally aligned fiber tracts. Patients’ reduced values for these parameters in the connection between left‐sided MFG and putamen/pallidum suggest an alteration of fiber integrity. It can be assumed that patients exhibit a loss of equally aligned and ordered fibers between MFG and subcortical structures of the basal ganglia. However, tractography does not provide any information about the fiber direction.

Anatomically, direct fibers connect cortical regions within the MFG with the putamen, an input nucleus of the basal ganglia. The reconstructed fibers between the MFG and pallidum are more difficult to interpret, as direct fiber tracts connecting both regions are anatomically not known. However, it should be taken into consideration that probabilistic fiber tract reconstruction does not reflect the real fiber structure in the brain, but rather a statistical illustration of possible structural connectivity. From this point of view, the findings can be interpreted as impaired structural connectivity between MFG and the basal ganglia system.

It is plausible that a modification of cortical input to the basal ganglia on the basis of structural alterations of fiber tracts could modulate activity/function within the basal ganglia network. Visually, less fiber tracts were displayed in patients between more caudal parts of the MFG, harboring the PMC, and the putamen. However, no statistical analyses regarding the fiber count were made because of its limited validity due to the supposed impact of individual characteristics in a small sample size. Instead, more appropriate diffusivity parameters such as FA and CL were statistically analyzed to characterize the fiber tracts as an illustration of structural connectivity. The statistical results showing lower mean FA values for patients’ fibers can be affirmed by fiber visualization in FA value coding (see Figure 2). It shows that these differences notably occur within the course of fibers between cortical areas, harboring PMC, and putamen, especially in the white matter directly adjacent to premotor cortical areas. Reconstructed fibers run between PMC and anterior parts of the putamen (see Figure 2). This is in line with the previously described rostrocaudal gradient of structural cortical connection in the striatum and the dominance of the rostral putamen in the connection to the PMC (Haber, 2016). Although these more detailed descriptive results lack statistical proof, they provide further information on the functional meaning of our findings. As a limitation of our analysis, the predefined ROI set in our study may underrepresent specific subregions of premotor and the primary sensorimotor cortices (e.g., hand areas) and therefore lowers specificity for detecting possible alterations of fiber tracts between those regions.

In an explorative approach, the structural alterations in corticostriatal connections found for the left hemisphere were also tested in the right hemisphere in a post hoc analysis. Hereby, no significant differences in fiber integrity were found between patients and controls. This supports the predominant involvement of left‐sided corticostriatal fibers in the pathophysiology of right‐sided WC. In consideration of the post hoc character of this analysis, the absence of structural alterations in the right‐sided corticostriatal connections cannot be interpreted as primary results, because they were not tested on the basis of a primary hypothesis. But they improve the validity of the structural connectivity differences found in corticostriatal connections in the left hemisphere by increasing the specificity and focus of alterations on the left hemisphere.

### 4.2 Interpretation of structural alterations in the context of existing pathophysiological concepts in focal dystonia

All regions of interest that we defined for our tractography analysis are known to exhibit changes in activation in task‐related fMRI (Blood et al., 2004; Castrop et al., 2012; Delmaire et al., 2005) and PET studies (Ceballos‐Baumann et al., 1997; Ibanez et al., 1999) in patients with WC. These activation changes have previously been interpreted mainly as hyperactivity due to reduced inhibition or underactivity sometimes discussed as reduced inhibitory neuronal activity. Also, the functional connectivity within the basal ganglia–thalamo–cortical loop seems to be changed in WC patients as previously shown in a resting‐state fMRI study (Dresel et al., 2014). This raises the question of a structural correlate underlying these functional alterations. Until now, the exact pathophysiological coherence between functional and structural imaging findings in brain networks is unclear, so that in the following we interpret structural alterations in the context of preexisting pathophysiological models in focal dystonia.

One of the main pathophysiological concepts is reduced inhibition in cortical–subcortical networks, resulting in a lack of movement restriction. Based on electrophysiological findings, it is assumed that “surround inhibition” (SI) allows the execution of precise movements by suppressing reverse stimuli (Quartarone & Hallett, 2013). Consequently, SI reduction could result in a defective planning of movements as shown in focal hand dystonia (Beck et al., 2008; Hallett, 2011; Sohn & Hallett, 2004). Different concepts exist to locate this imbalance between excitation and inhibition within the motor system. The basal ganglia modulate motor programs via direct and indirect basal ganglia–thalamo–cortical pathways (Mink, 1996). Here, a lack of cortical inhibition in dystonia could secondarily arise by overactivity of the direct pathway or underactivity of the indirect pathway (Hallett, 2006). In this context, reduced structural connectivity between PMC and putamen in our study could result in altered cortical input to the basal ganglia system that in the following disturbs the activity within the just mentioned basal ganglia–cortical loops and in the end affects movement selection and inhibition on the subcortical and cortical level.

Our structural alterations match up previous functional findings supporting a functional role of the PMC–basal ganglia connections in focal dystonia. Transcranial magnetic stimulation (TMS) studies showed the involvement of cortical regions such as PMC in the just mentioned SI (Beck, Houdayer, Richardson, & Hallett, 2009). Electroencephalography and electromyography measurements pointed out that repetitive transcranial magnetic stimulation (rTMS) of the lateral PMC leads to a transient reorganization of neuronal activity in cortical sensorimotor areas (Chen et al., 2003). For patients with focal arm dystonia, an increased sensitivity to slow‐frequency rTMS of the dorsal PMC was shown by PET and has been interpreted as increased modifiability of the motor system in dystonia (Siebner et al., 2003). Reduced excitability of inhibitory systems contributes to those findings (Filipovic et al., 1997; Ikoma, Samii, Mercuri, Wassermann, & Hallett, 1996; Ridding, Sheean, Rothwell, Inzelberg, & Kujirai, 1995). Corresponding to that, decreased GABA levels were observed in the sensorimotor cortex and lentiform nuclei, by use of MR spectroscopy in WC (Levy & Hallett, 2002). Functional findings using PET (Ceballos‐Baumann & Brooks, 1998; Ceballos‐Baumann et al., 1997; Ibanez et al., 1999) or fMRI (Castrop et al., 2012; Delnooz, Helmich, Medendorp, Warrenburg, & Toni, 2013; Gallea, Horovitz, Ali Najee‐Ullah, & Hallett, 2016) showed changes in activation within the PMC in patients with WC. Another PET study described a reduced PMC activation as well as decreased functional correlation between PMC and putaminal activity (Ibanez et al., 1999). It can be supposed that the impaired structural connectivity that we show between PMC and putamen correlates with an altered premotor and basal ganglia activity, triggered by the imbalance between excitation and inhibition in cortical–subcortical networks.

Impaired inhibition at the cortical and subcortical level could result in altered neuronal plasticity (Lin & Hallett, 2009), as a further pathophysiological mechanism in dystonia (Hallett, 2011). In this context, repetitive writing movements could lead to a maladaptive plasticity due to a deficient adaptation (Weise et al., 2006). A disturbance of this homeostatic‐like plasticity that controls cortical excitability is assumed in focal hand dystonia (Quartarone et al., 2005). Such abnormal plasticity in combination with environmental factors like frequent repetitive movements lead to the two‐factor hypothesis in the pathophysiology of dystonia (Quartarone & Hallett, 2013). In concrete terms, an abnormality of the normal homuncular organization of the finger representation was shown in the somatosensory cortex for focal hand dystonia (Bara‐Jimenez, Catalan, Hallett, & Gerloff, 1998). Our result of reduced structural connectivity between PMC and basal ganglia may contribute to a maladaptive plasticity within cortical and subcortical sensorimotor areas. The impaired integrity of the fibers linking PMC to the putamen as an input nuclei of the basal ganglia system could be associated with plasticity changes within the subcortical gray matter structures. A disorganized somatotopic representation of individual fingers has been shown in the putamen in focal hand dystonia (Delmaire et al., 2005). Together with the mechanisms of reduced inhibition and selection of motor programs mentioned above, this could secondarily affect cortical sensorimotor plasticity.

In summary, the demonstrated impairment of structural connectivity between the PMC, as a location for movement planning, and the basal ganglia system, where motor programs are selected, can be well integrated into the pathophysiologic ideas of reduced inhibition and maladaptive sensorimotor plasticity at the basal ganglia and cortical level. At the end, our results could reflect a structural correlate contributing to the functional selectivity loss of muscle activation in focal hand dystonia by a dedifferentiation of sensorimotor programs (Quartarone & Hallett, 2013).

## DISCLOSURES

A. Jochim has received travel grants from The International Parkinson and Movement Disorder Society, Ipsen Pharma GmbH, Merz Pharmaceuticals GmbH, Pharm‐Allergan GmbH, AbbVie Deutschland GmbH & Co. KG, Boston Scientific, Medtronic GmbH, and Universitätsklinikum Würzburg, as well as speaker honoraria from Pharm‐Allergan GmbH. B. Haslinger receives research support from the German Research Foundation (DFG) and Ipsen. Y. Li has no financial disclosures. G. Gora‐Stahlberg has no financial disclosures. M. Berndt has no financial disclosures.
