# Supplementary material for: Impaired white matter integrity between premotor cortex and basal ganglia in writer’s cramp
Source: Brain Behav. 2018 Sep 21;8(10):e01111. doi: 10.1002/brb3.1111 (PMC6192408; doi:10.1002/brb3.1111)
Supplement: Supplementary file 1 [file BRB3-8-e01111-s001.docx]

**Impaired white matter integrity between premotor cortex and basal ganglia in writer´s cramp**

Maria Berndt^1,2^, Yong Li^1^, Gina Gora-Stahlberg^1^, Angela Jochim^1^, Bernhard Haslinger^1^

(1) Department of Neurology, Klinikum rechts der Isar, Technische Universität Muenchen, Muenchen, Germany

(2) Department of Neuroradiology, Klinikum rechts der Isar, Technische Universität Muenchen, Muenchen, Germany

**Supplement**

**Statistical parameters of the CL-analysis**

| Connection | CL controls |  | CL patients |  | p-Value |
| --- | --- | --- | --- | --- | --- |
| MFG L - | Mean | SD | Mean | SD | (*=significant after correction) |
| Putamen L | 0.384 | 0.031 | 0.355 | 0.023 | 0.003* |
| Pallidum L | 0.400 | 0.018 | 0.363 | 0.021 | 0.00001* |
| Caudate L | 0.358 | 0.044 | 0.345 | 0.037 | 0.529 |
| Thalamus L | 0.398 | 0.026 | 0.380 | 0.032 | 0.134 |
| Precentral L | 0.386 | 0.038 | 0.377 | 0.019 | 0.337 |
| Postcentral L | 0.381 | 0.031 | 0.376 | 0.032 | 0.663 |

Table 1: Mean and standard deviation (SD) of the linear anisotropy (CL) for the tested connections with the middle frontal gyrus (MFG) and p-values of the mean value comparison

**Post hoc analysis**

Based on the results in the left hemisphere, a post hoc analysis was performed to investigate the right sided homologous tracts between the middle frontal gyrus and the putamen as well as the pallidum. Only very small differences in the FA values were found between patients and controls, shown in the following Table 2. The p-values of mean value comparisons between patients and controls (two-sample t-test for independent samples) showed no statistical significance.

| **Connection** | **FA controls** |  | **FA patients** |  | **p-Value** |
| --- | --- | --- | --- | --- | --- |
| MFG R - | Mean | SD | Mean | SD |  |
| Putamen R | 0.396 | 0.020 | 0.386 | 0.031 | 0.314 |
| Pallidum R | 0.413 | 0.042 | 0.395 | 0.029 | 0.201 |

*Table 2: Mean and standard deviation (SD) of the fractional anisotropy (FA) for the right-sided connections between middle frontal gyrus (MFG) and putamen/pallidum and p-values of the mean value comparison*
